# Heart Failure in Type 1 vs. Type 2 Diabetes Mellitus: Shared Pathways, Distinct Challenges

**DOI:** 10.1155/jdr/7460084

**Published:** 2026-05-03

**Authors:** Muhammad S. Hussain, Saraswathi Iyer, Yi Jia Liew, Mya Lelt Win, Rory J. McCrimmon, Chim C. Lang, Ify R. Mordi

**Affiliations:** ^1^ Division of Cardiovascular Research, University of Dundee, Dundee, Scotland, UK, dundee.ac.uk; ^2^ Division of Diabetes, Endocrinology and Reproductive Biology, University of Dundee, Dundee, Scotland, UK, dundee.ac.uk

**Keywords:** cardiac microvascular dysfunction (CMD), GLP-1RA and finerenone, heart failure (HF), HFpEF, HFrEF, SGLT-2i inhibitors and Sotagliflozin, type 1 diabetes (T1D), type 2 diabetes (T2D)

## Abstract

Heart failure (HF) is a critical complication in both type 1 (T1D) and type 2 diabetes (T2D) and people with diabetes are at higher risk of developing HF than those without diabetes. The pathophysiology of HF in diabetes often involves diabetic cardiomyopathy, driven by insulin deficiency, insulin resistance (IR), inflammation, and myocardial fibrosis; however, though there are similarities in HF in T1D and T2D, there are also key differences in epidemiology, pathophysiology, treatment, and clinical outcomes. In this review article we will discuss the burden, pathophysiology, and outcomes of HF in diabetes, focusing on differences between T1D and T2D, and the relative unmet need for patients with T1D and HF.

## 1. Introduction

Heart failure (HF) is a complex clinical condition characterized by the heart’s impaired ability to pump sufficient blood to meet the body’s metabolic demands. This arises from underlying structural alterations in the heart, such as myocardial hypertrophy and ventricular dilatation, alongside functional disturbances including impaired myocardial relaxation and reduced contractility [1]. Both types of HF, HF with preserved ejection fraction (HFpEF) and HF with reduced ejection fraction (HFrEF) entail a poor prognosis, despite the advances in pharmacological treatments over the past decade [2–4]

Patients with HF experience high morbidity, mortality, and recurrent hospitalizations. Globally, over 1% of the population is affected, and the prevalence rises sharply with age [5]. In one Dutch study, HF prevalence was 11.8% of individuals aged ≥60 years, corresponding to 4.2% of the general population [6]. In the UK, primary care data (Conrad et al. [7]) from ~4 million individuals identified a national HF prevalence of 1.6%. The incidence and prevalence of HF are expected to increase further due to aging populations and improved survival after cardiovascular events such as myocardial infarction (MI) [7]. Although HF predominantly affects older adults, its incidence is increasing among individuals under 50 years of age, driven by rising rates of obesity, hypertension, atrial fibrillation, and, notably, diabetes mellitus [8]. Mortality and hospitalization rates remain high despite advances in management, with 2‐year mortality around 20%–30% even in clinical trials involving closely monitored participants. In a recent Turkish study, the 5‐year mortality was up to 50%, and ~40% of new HF patients do not survive beyond the first post diagnosis [9, 10]. HF is the leading cause of hospitalization in individuals over 65, with nearly 50% readmitted within the first year and 20%–25% experiencing multiple admissions, often due to cardiovascular or comorbid conditions such as diabetes [11, 12].

Diabetes mellitus is a major contributor to HF risk and is linked to adverse clinical outcomes. Approximately 35%–45% of individuals with diabetes also have HF [13]. Diabetes increases both mortality and hospitalization rates in patients with HF. In an UK observational study of 87,709 HF patients with comorbid T2D and chronic kidney disease (CKD) over 20 years, hospitalization rates were highest among those with CKD and T2D. Median survival was longest in the reference group (4.4 years) and in HF‐T2D‐only patients (4.1 years), but markedly lower in HF‐CKD‐only patients (2.2 years), highlighting CKD as a key driver of poor outcomes requiring targeted intervention [14].

Poor glycaemic control is a well‐recognized, independent risk factor for HF in both T1D and T2D, contributing to diabetic cardiomyopathy, a condition characterized by structural and functional changes in the heart muscle independent of coronary artery disease or hypertension [15]. While both T1D and T2D increase HF risk, the underlying pathophysiology may differ, influencing disease progression and treatment outcomes. This review provides a comparative analysis of HF in T1D and T2D, highlighting how their distinct mechanisms—such as glucotoxicity and insulin deficiency versus lipotoxicity and systemic inflammation—lead to unique clinical phenotypes, divergent outcomes, and specific challenges in therapeutic management.

## 2. Epidemiology

**Figure 1:**
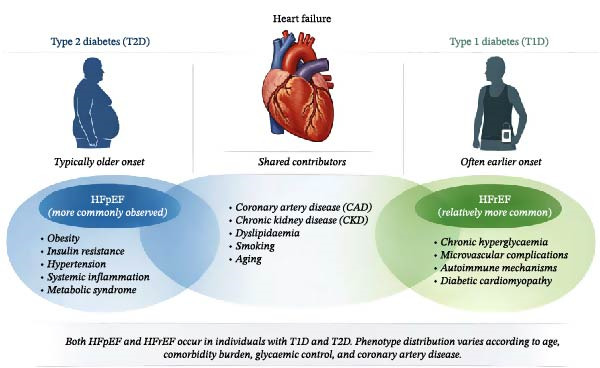
Comparative schematic of heart failure in type 1 and type 2 diabetes. The figure contrasts the typical age of onset, predominant heart failure phenotype (HFrEF vs. HFpEF), and primary pathophysiological drivers between the two populations.

The key epidemiological and pathophysiological differences between HF in T1D and T2D are summarized in Figure 1. T2D dominates globally, with 462 million cases (6.28% of the population) and disproportionately high cardiovascular risk [16]. In the UK, 4.6 million people have diagnosed T2D, with 5.8 million total living with diabetes, including 1.3 million undiagnosed [17]. In contrast, T1D is less prevalent, comprising 9.5% of the global diabetes population. In the UK, around 400,000 people are living with T1D, including ~29,000 children [18]. Although HF is typically a disease of aging, and the absolute burden of HF is substantially higher in T2D due to older age at diagnosis, overall disease prevalence and the presence of comorbidities such as hypertension, obesity, and dyslipidaemia, HF actually represents a significant issue in T1D also. Individuals with T1D face a disproportionately elevated relative risk as highlighted by epidemiological data. Avogadro et al. [19] reported that the risk of HF in individuals with type 1 diabetes (T1D) was three times higher than in the general population. Kristófi et al. [20] further demonstrated that this elevated risk persists even when compared to type 2 diabetes (T2D), after adjusting for age and comorbidities. These findings are supported by a Scottish cohort study by McAllister et al. [21], which included 3.25 million adults over the age of 30 and found that HF hospitalizations were more than twice as common in people with diabetes compared to those without (15 vs., 11 per 1000 person‐years). Within the diabetic cohort, T2D had higher HF incidence, reflecting larger population size and comorbidity burden, while T1D patients were hospitalized roughly 20 years younger, indicating longer lifetime burden [21]. Gender modifies this risk, as diabetes is associated with a twofold increase in HF risk in men and a fivefold increase in women relative to non‐diabetics [22]. Studies have also highlighted that regions with more ethnic minority populations, hypertension, heart disease, and older age show the strongest associations with diabetes prevalence [23].

Recent data indicate that adults with T1D face a markedly elevated HF risk relative to those with T2D when age stratified. In a two‐country registry study of 59,331 T1D and 484,241 T2D individuals, the hazard ratio (HR) for HF in T1D vs T2D was ~1.3–1.4 in the 65–79‐year age group [20]. Similarly, a meta‐analysis of 61,885 individuals with T1D, 248,021 with T2D, and over 4.5 million non‐diabetic controls reported HF incidence rates of 5.8 and 10.0 per 1000 person‐years in T1D and T2D, respectively; when compared with non‐diabetics, T1D was associated with an adjusted relative risk (aRR) of 3.4 (95% CI 2.71–4.26) [24]. A French nationwide cohort of hospitalized patients further showed a ~1.4‐fold higher HF risk in T1D compared with T2D among those aged >60 years. HF risk in T1D may be under‐recognized because most HF trials focus on T2D, with T1D individuals rarely included [25]. HF prevalence in diabetes to be as high as 22% and reports a 2–4‐fold increased HF risk in both T1D and T2D compared to those without diabetes [26].

Prognostic differences are also evident. After HF hospitalization, 30‐day mortality is significantly higher in T1D than in T2D, even after adjustment for age, sex, and socioeconomic status [13]. Scandinavian registry data corroborate these findings, showing higher HF incidence and cardiovascular mortality in T1D compared with T2D despite adjustment for confounders [27]. In contrast, 1‐year mortality following HF hospitalization in T2D ranges from 40% to 50%, increasing further in those with multiple comorbidities or CKD [12].

The impact of diabetes on HF outcomes also varies by age. Evidence supports a significant age–diabetes interaction, where diabetes confers a greater relative mortality risk in younger individuals with HF. Specifically, diabetes strongly predicts 5‐year mortality in HF patients aged <75 years (*p* < 0.001), while this association is attenuated and non‐significant in those ≥75 years (*p* = 0.16), suggesting that diabetic cardiomyopathy may be particularly deleterious when manifesting earlier in life [28].

Undiagnosed HF is another challenge in diabetes. Boonman‐de Winter et al. [29] reported that up to 28% of people with T2D had undiagnosed HF, often HFpEF. Data on this for T1D remain limited, suggesting an under‐recognition in this population.

Both diabetes and HF impose a significant financial burden on healthcare systems. Diabetes is associated with reduced life expectancy, frequent hospital admissions, longer inpatient stays, and higher care requirements. NHS England reported that the cost of diabetes medications alone was approximately £1 billion during 2017–2018. Moreover, the presence of diabetes‐related comorbidities such as cardiovascular disease (CVD) and cancer, further strains the system, contributing an additional estimated £3 billion to the NHS budget annually [30]. According to the National Diabetes Inpatient Audit, individuals with diabetes account for 18% of hospital bed occupancy, compared with 7% among those without diabetes [31]. HF similarly places a heavy strain on healthcare systems. In the UK, this condition accounts for nearly one million inpatient bed days annually, representing ~2% of NHS inpatient capacity and 5% of emergency medical admissions [32]. Globally, an estimated 64.3 million individuals live with HF. In the United States, in 2014, HF was responsible for 1.1 million emergency department visits, 980,000 hospitalizations, and 84,000 primary deaths; these figures increased fourfold when HF was a contributing comorbidity [33]. The estimated cost of these healthcare contacts in 2014 was approximately $11.3 billion (~$11,500 per patient per hospitalization), and these figures have almost certainly increased since then.

The financial burden of HF in individuals with T1D is relatively underexplored but is likely to be substantial given the younger age at onset of HF in T1D, which results in longer cumulative exposure to morbidity and increasing complexity [21, 34]. Although the prevalence of T2D drives aggregate costs into the billions, the per‐patient economic burden in T1D is considerable due to earlier onset of complications and prolonged disease duration, beyond the costs of insulin and glucose monitoring technologies alone. For example, with a 3.1% HF prevalence among ~1.9 million individuals with T1D in the US, HF hospitalizations alone cost an estimated $29 million annually [35]. In the UK, diabetes‐related costs to the National Health Service (NHS) total approximately £14 billion annually, including over £10 billion in direct healthcare expenses. Preventable complications such as HF contribute around £6.2 billion of this sum yearly [36]. Hospital costs linked to cardiac events are notably higher per patient in T1D, averaging an additional £149 per year compared with people with T2D after adjustment for confounders [23]. These figures highlight that, despite representing a smaller proportion of the diabetes population, individuals with T1D incur disproportionately high HF‐related healthcare costs.

While the economic burden of diabetes and HF is well‐characterized in high‐income countries (HICs), data from low and middle‐income countries (LMICs) remain scarce. This represents a critical evidence gap, as LMICs shoulder over 75% of the global CVD mortality and are experiencing a rapid rise in diabetes prevalence, projected to increase by 134% by 2045 [37, 38]. A systematic review of HF costs in LMICs found that direct medical costs accounted for 31%–88% of household income, indicating catastrophic health expenditure, however, such analyses are limited to only a handful of countries [39].

## 3. Pathophysiology

While both T1D and T2D substantially increase the risk of HF, this risk arises from distinct yet overlapping pathophysiological mechanisms, as summarized in Table 1. Although traditional cardiometabolic risk factors contribute in both conditions, the relative impact of poor glycaemic control is particularly pronounced in T1D, where it is a primary driver of cardiomyopathy [40]. These differing origins, absolute insulin deficiency in T1D versus insulin resistance (IR) in T2D, give rise to divergent inflammatory, fibrotic, and microvascular pathways that ultimately converge on myocardial dysfunction.

**Table 1:** Comparison table: heart failure mechanisms in type 1 vs type 2 diabetes.

Category	T1D (Type 1 diabetes)	T2DM (Type 2 diabetes mellitus)	Shared pathways & outcomes
Primary insulin issue	Autoimmune destruction of β‐cells → Absolute insulin deficiency	Peripheral insulin resistance and relative insulin deficiency	Result: Chronic hyperglycemia
Prevalence of HF type	Less defined; often HFrEF (reduced ejection fraction)	~ 50% of HF cases are HFpEF (preserved ejection fraction)	Heart failure risk present in both
Triggering comorbidities	Often fewer metabolic comorbidities	Obesity, hypertension, dyslipidaemia, metabolic syndrome	Metabolic comorbidities worsen cardiac function
Inflammatory response	Less prominent; autoimmune inflammation more systemic	Chronic low‐grade inflammation → ↑ TNF‐α, IL‐6	Drives myocardial remodeling and fibrosis
Oxidative stress mechanism	Indirect via chronic hyperglycemia	Exacerbated by adiposity, mitochondrial overload	Leads to cellular injury and fibrosis
Structural heart changes	Less common unless poorly managed	LV hypertrophy, diastolic stiffness, cardiac steatosis	Contributes to diastolic dysfunction
RAAS & neurohormonal activation	Secondary to glucose dysregulation	More pronounced due to volume/pressure overload	Promotes fibrosis, sodium retention, HF progression
End result	HF (often HFrEF) through shared damage pathways	Predominantly HFpEF, especially in obese/older patients	↑ Cardiovascular morbidity and mortality

In T2D, around half of all HF cases present as HFpEF, largely driven by prevalent comorbidities such as obesity, hypertension, and IR, which exert a strong additive effect on HF risk [41, 42]. These metabolic comorbidities promote atherosclerosis, coronary microvascular disease, left ventricular hypertrophy (LVH), increased myocardial stiffness, and diastolic dysfunction, thereby predisposing to HFpEF [43]. In contrast, in T1D, HF pathogenesis has been more closely linked to chronic hyperglycemia, microvascular dysfunction, advanced glycation end products, and autonomic neuropathy, leading to direct diabetic cardiomyopathy [44]. That said, the historical view of T1D as a lean phenotype is changing, increasing obesity rates among people with T1D are narrowing the phenotypic gap, meaning that overweight or obese T1D patients may increasingly share the augmented HF risk conferred by metabolic comorbidities [25].

Regarding inflammation, T2D is characterized by adipose tissue‐derived low‐grade inflammation, where fat acts as an endocrine organ, secreting pro‐inflammatory cytokines like TNF‐α and IL‐6 into the circulation, which directly promote myocardial inflammation, endothelial dysfunction, and cardiac IR. This inflammation in HF with T2D, contributes to progressive cardiac dysfunction [45, 46]. In contrast, the inflammatory state in T1D is more secondary to hyperglycemia (glucotoxicity). Inflammatory activation in T1D likely reflects both autoimmune mechanisms and metabolic stress, whereby hyperglycemia and ROS trigger NF‐κB–mediated cytokine release, fibroblast recruitment, and pro‐fibrotic signaling typical of diabetic cardiomyopathy, resulting in low‐grade myocardial inflammation [47].

In T2D, cardiac fibrosis is heavily promoted by lipotoxicity. The overflow of fatty acids to the heart leads to the accumulation of toxic lipid intermediates (e.g., ceramides and diacylglycerols), which directly trigger pro‐fibrotic signaling (e.g., TGF‐β) and generate substantial oxidative stress from mitochondrial overload and inefficient fatty acid oxidation [48]. Increased free fatty acid influx and IR further drive excessive mitochondrial production of reactive oxygen species (ROS), AGE‐RAGE signaling, and NADPH oxidase activation, which promote oxidative stress and interstitial fibrosis in the myocardium [49]. Visceral fat accumulation contributes to ectopic lipid deposition in the heart (cardiac steatosis), impairing both systolic and diastolic function. IR in T2DM, particularly in cardiac and skeletal muscle, disrupts normal glucose uptake, shifting myocardial energy substrate preference from glucose to fatty acids [50, 51]. This shift may contribute to reduced ATP generation, increased oxygen consumption, and mitochondrial dysfunction [52].

These mechanisms create a cycle where lipid‐induced oxidative stress damages cardiomyocytes and promotes interstitial fibrosis. Conversely, in T1D, fibrosis and oxidative stress are predominantly initiated by glucotoxicity. Constant hyperglycemia fuels the polyol pathway and protein kinase C (PKC) activation, which are potent stimulators of NADPH oxidase and a major source of superoxide production. This “glucose‐induced” oxidative stress, along with AGE‐mediated cross‐linking of collagen, is a primary instigator of myocardial fibrosis in the insulin‐deficient heart [53]. Therefore, the fibrotic remodeling of the heart in T2D is a result of lipid overload, while in T1D, it is a result of glucose toxicity and associated oxidative pathways.

Collectively, these mechanisms favor HFpEF, commonly seen in T2D, and exacerbate overall cardiovascular risk [54]. Table 1 presents a side‐by‐side comparison of heart failure mechanisms in T1D versus T2D along with shared pathways and possible outcomes.

A unifying mechanism underlying these processes is endothelial dysfunction, which represents a pivotal early event in the development of HF in diabetes. Impaired endothelial nitric oxide (NO) production leads to reduced vasodilation, increased vascular stiffness, and promotes a pro‐inflammatory and pro‐thrombotic state. These changes exacerbate myocardial ischemia and fibrosis, further impairing cardiac function. Thus, endothelial dysfunction acts as a crucial link between metabolic disturbances and structural cardiac abnormalities in both T1D and T2D [55].

In contrast to T2D, HF in T1D was thought to predominantly present as early‐onset systolic dysfunction and may arise independently of traditional cardiovascular risk factors [19]. The pathophysiology of HF in T1D appears to involve distinct mechanisms, including autoimmune injury, prolonged hyperglycemia, and chronic insulin therapy [34]. Traditionally it has been thought that the majority of HF in T1D is due to the elevated risk of MI, which can lead to HF through ischemic pathways. However, many T1D individuals develop HF symptoms in the absence of a MI, suggesting alternative mechanisms such as chronic hyperglycemia and diabetic cardiomyopathy.

In some individuals, protective cardiac adaptations may delay the onset of heart failure. These findings point to a unique resilience in some individuals and highlight the need for further research into the mechanisms behind this cardioprotection [56]. Poorly managed T1D shows a stark contrast: even a 1% increase in HbA1c increases the risk of HF by 30%, surpassing the risk seen in individuals with IR, T2D mellitus (T2DM), and HbA1c levels over 8% [57]. Findings from the Diabetes Control and Complications Trial (DCCT), a 6.5‐year prospective study, demonstrated that intensive insulin therapy reduced HF incidence compared to conventional therapy (7.2% vs 9.1%) [58]. The follow‐up Epidemiology of Diabetes Interventions and Complications (EDIC) study further confirmed a direct relationship between HbA1c and HF risk in T1D, revealing that each 1% increase in HbA1c was associated with a 3.5‐fold rise in HF incidence [59].

Cardiac autoimmunity may contribute to HF pathogenesis in T1D. The DCCT/EDIC study found that, patients positive for two or more cardiac‐specific autoantibodies, such as anti‐myosin and anti‐troponin, exhibited increased LV mass, reduced ejection fraction, and higher rates of HF, particularly among those with poor glycaemic control [58, 60]. These findings support the role of direct immune‐mediated myocardial injury as a non‐ischemic pathway of systolic dysfunction in T1D [61]. As highlighted by Sousa et al. [62], such autoimmune mechanisms may explain the disproportionate HF burden in T1D, even in the absence of traditional risk factors

In T1D, microvascular dysfunction is similarly implicated in HF pathogenesis through mechanisms such as basement membrane thickening and endothelial cell damage in myocardial capillaries. This reduces myocardial capillary density and impairs nutrient and oxygen delivery, promoting cardiomyocyte injury and fibrosis. Clinical correlates such as diabetic retinopathy and nephropathy often parallel cardiac microvascular disease, suggesting systemic microangiopathy significantly influences HF risk and progression in people with T1D [63]. In T1D, prevalent microvascular complications, including retinopathy, nephropathy, and autonomic neuropathy, contribute to volume overload, increasing right‐ and left‐sided cardiac pressures and accelerating progression to congestive HF (CHF) [44, 64]. Poor glycaemic control further exacerbates microvascular injury, resulting in earlier and more severe HF. Renal impairment in T1D generates a feedback loop characteristic of cardiorenal syndrome, whereby worsening kidney function intensifies cardiac congestion and vice versa [65]. Incorporating microvascular diagnosis in T1D improves diagnostic accuracy and facilitates timely management.

Molecular studies highlight the role of inflammation in the development of diabetic cardiomyopathy and cardiac hypertrophy in T1D. Endothelial cell activation leads to dysfunction of endothelial NO synthase (NOS), reducing NO production. This decreases cyclic guanosine monophosphate (cGMP) levels in myocardial cells, impairing ATP generation, which is essential for myocardial contraction and relaxation. ATP deficiency further contributes to mitochondrial dysfunction and increases ROS and superoxide production, resulting in myocardial damage, inflammation, LV hypertrophy, and eventually HF [32, 33]. Another proposed mechanism involves impaired calcium accumulation due to reduced calcium pump activity, linked to low expression of glucose transporter type 4 (GLUT4) in cardiomyocytes. This disruption in calcium homeostasis compromises myocardial contractility and contributes to cardiomyopathy [7].

Additional factors such as age, IR, and microvascular complications also contribute to HF risk. Emerging evidence indicates that systemic microvascular disease plays a key role in HF development, with markers such as diabetic retinopathy and albuminuria being associated with impaired myocardial flow reserve. These associations highlight the importance of cardiac microvascular dysfunction (CMD) in the pathophysiology and clinical outcomes of HF in individuals with T1D [66].

CMD is increasingly recognized as a key contributor to the pathogenesis of diabetic cardiomyopathy and HF [67]. CMD is characterized by impaired myocardial perfusion resulting in structural and functional abnormalities in the small vessels supplying the myocardium, including capillaries and arterioles. This compromised microcirculatory function leads to myocardial ischemia, interstitial fibrosis, and diminished cardiac contractility, ultimately reducing cardiac output. In both T1D and T2D, CMD predominantly affects the coronary microcirculation, capillaries, and small arterioles supplying the myocardium. Unlike obstructive coronary artery disease, CMD involves impaired blood flow in the absence of significant large‐vessel stenosis, a condition often referred to as microvascular angina. Histopathological studies from ventricular myocardial biopsies in people with diabetes demonstrate thickening of the capillary basement membrane and interstitial fibrosis, providing clear evidence of microvascular damage within the myocardium [68].

In T2D, CMD is closely linked to classic cardiometabolic risk factors such as obesity, IR, hypertension, and systemic inflammation. These factors contribute to endothelial dysfunction and microvascular rarefaction, promoting myocardial stiffening and LVH. Clinically, people with T2D often present with HFpEF, characterized by diastolic dysfunction alongside preserved systolic function. Additionally, neurohormonal imbalances, particularly overactivation of the renin–angiotensin–aldosterone system (RAAS) and sympathetic nervous system (SNS), further exacerbate microvascular impairment and disrupt glucose homeostasis, perpetuating a vicious cycle that exacerbates both cardiac and metabolic dysfunction [69, 70].

In contrast, CMD in T1D may arise more directly from chronic hyperglycemia and autoimmune‐mediated vascular injury, rather than broader cluster of metabolic syndrome components typically seen in T2D. Although mechanisms such as endothelial dysfunction and oxidative stress are common to both T1D and T2D, the persistent hyperglycemia and autoimmune pathology characteristic of T1D may lead to earlier and more pronounced myocardial fibrosis and systolic dysfunction. Unlike T2D, where IR plays a central role, T1D individuals, particularly those with poor glycaemic control, are prone to significant microvascular complications, including retinopathy and nephropathy. These complications are closely correlated with cardiac microvascular impairment and an increased risk of HF [68, 71].

Hong et al. [33] highlighted a significant overlap between overt CMD and cardiac diastolic dysfunction, with both independently predicting adverse cardiovascular outcomes such as hospitalization for HF and cardiovascular mortality. This association underscores the prognostic significance of CMD in diabetes‐related HF, irrespective of diabetes type [71].

The diagnosis of CMD has evolved from a histopathological finding to a clinically assessable condition through advanced imaging. The reference standard remains invasive coronary reactivity testing, which assesses changes in coronary blood flow in response to pharmacological stimuli (e.g., acetylcholine) but is limited to specialized centers [72]. Non‐invasively, cardiac magnetic resonance (CMR) is the cornerstone, allowing for quantitative assessment of myocardial perfusion reserve (MPR) and extracellular volume (ECV)—a direct marker of interstitial fibrosis [73]. Positron emission tomography (PET) provides highly accurate quantitative myocardial blood flow measurements and is considered a non‐invasive reference for flow quantification [74]. In routine echocardiography, the assessment of coronary flow velocity reserve (CFVR) in the left anterior descending artery via Doppler is a feasible, though operator‐dependent, method [75]. Notably, a reduced global longitudinal strain (GLS) on speckle‐tracking echocardiography often correlates with underlying CMD, serving as a functional surrogate [76].

## 4. Diagnosis and Prevention Strategies

HF diagnosis in people with diabetes, whether T1D or T2D, begins with clinical assessment. Typical symptoms include breathlessness, orthopnoea, reduced exercise tolerance, fatigue, ankle swelling, and nocturnal cough. Patients with these symptoms should undergo preliminary investigations, including BNP/NT‐proBNP measurement, ECG, chest X‐ray, and blood tests for cardiac and renal function. Natriuretic peptides reflect ventricular stress and volume overload. Levels above diagnostic thresholds (BNP >35 pg/mL or NT‐proBNP >125 pg/mL) warrant echocardiography to confirm diagnosis and classify HF type. It is important to note, however, that NT‐proBNP levels below the diagnostic cutoff may still indicate subclinical dysfunction in high‐risk diabetic patients [77]. No T1D‐specific thresholds have been established.

The diagnostic accuracy of NP is not independently altered by diabetes itself. However, it can be confounded by comorbidities highly prevalent in diabetic populations, particularly obesity and CKD [78]. Obesity, more prevalent in T2D, tends to lower circulating BNP and NT‐proBNP levels, potentially through increased adiposity and altered adipokine signaling. In contrast, CKD elevates natriuretic peptide levels due to impaired renal clearance [79, 80]. Moreover, in T2D, symptoms such as dyspnea and edema may be misattributed to deconditioning or comorbidities like chronic obstructive pulmonary disease (COPD), creating diagnostic ambiguity [81]. In T1D, HF may be overlooked as symptoms such as fatigue and reduced exercise tolerance are often attributed to glycaemic variability rather than prompting early cardiac evaluation [34]. Clinicians must therefore interpret NP results in the context of comorbidities and clinical presentation to avoid both under‐ and over‐diagnosis.

Importantly, hyperglycemia or diabetes are not established independent modifiers of NP accuracy. Therefore, while standard diagnostic thresholds (e.g., NT‐proBNP >125 pg/mL) remain applicable, results must be interpreted in the context of body habitus and renal function. Integrating NP measurements with clinical assessment and cardiac imaging remains essential for accurate HF diagnosis and risk stratification.

In addition to natriuretic peptides, other biomarkers may have incremental value in early detection of diabetic cardiomyopathy. In diabetic cohorts, elevated high‐sensitivity cardiac troponin (hs‐cTnT), even within the “normal” range, may be a predictor of future HF; one study found patients with levels ≥14 ng/L had a 4.3‐fold higher risk of developing [82]. Similarly, soluble ST2 (sST2), which reflects cardiac fibrosis, may have additional prognostic value, with levels >35 ng/mL being associated with a >50% increased risk of HF hospitalization or cardiovascular death [83]. The combination of these markers may provide a multi‐faceted assessment (stress, injury, fibrosis) and may improve early risk stratification beyond a single marker, though further validation is required.

In patients with suspected HF, echocardiography is essential to establish the diagnosis and to classify disease according to left ventricular ejection fraction (LVEF). This enables differentiation between HFrEF (LVEF <40%), HFmrEF (mildly reduced ejection fraction; LVEF 41%–49%) and HFpEF (preserved ejection fraction; LVEF ≥ 50%) [1]. Accurate phenotyping is essential to guide personalized management and risk stratification, particularly in patients with diabetes, who are susceptible to all HF subtypes due to shared metabolic and cardiovascular pathophysiology.

HF subtypes differ in both pathophysiological mechanisms and therapeutic responses. HFmrEF represents a heterogeneous intermediate group, sharing characteristics of both HFrEF and HFpEF [15]. A patient‐level, pooled meta‐analysis of DAPA‐HF and DELIVER indicates that therapies such as SGLT2 inhibitors (dapagliflozin) reduce cardiovascular death or worsening HF across the EF spectrum in both diabetic and non‐diabetic patients. Epidemiological studies suggest that HFmrEF is overrepresented in diabetic populations (25.4% vs 16.7% in non‐diabetics) [84].

Diabetic cardiomyopathy typically begins with left ventricular diastolic dysfunction, progresses to subtle subclinical systolic impairment with preserved ejection fraction, and eventually advances to HFrEF. Diastolic dysfunction is widely recognized as the earliest echocardiographic manifestation in both T1D and T2D, and emerging evidence highlights the role of left atrial dysfunction, such as reduced left atrial phasic strain, as an early and sensitive marker. Ifuku et al. [85] observed impaired left atrial function in adolescents with T1D (*n* = 53) compared to non‐diabetic controls (*n* = 53), supporting its value in identifying early diastolic impairment. The *E*/*e*′ ratio is frequently used to estimate diastolic filling pressures, with studies reporting higher values in T1D patients compared to controls, although findings are not entirely consistent [86]. Kaushik et al. [87] identified preclinical ventricular changes detectable by echocardiography in young individuals with T1D. They observed reduced left ventricular strain across multiple segments, basal lateral, mid‐lateral, basal septum, and mid‐septum, compared with non‐diabetic peers, despite normal ejection fraction and absence of overt HF symptoms [87]. Additionally, endothelial function measured by flow‐mediated dilatation (FMD) was significantly lower in the T1D group. These changes correlated with HbA1c levels, indicating that chronic hyperglycemia may play a role in early myocardial impairment. Detecting these subclinical regional dysfunctions through deformation imaging, such as strain and strain rate analysis, may serve as an effective method for identifying asymptomatic diabetic patients at risk.

Left ventricular diastolic dysfunction is widely recognized as one of the earliest detectable signs of HF in individuals with diabetes. In the Thousand & 1 Study of 960 patients with preserved LVEF (≥45%) and median 6.3 years follow‐up, 12% experienced MACE. Elevated diastolic function marker E/e′ ratio showed a strong association with MACE risk (adjusted HR 4.29 per log increase), outperforming NT‐proBNP (HR 1.56), and combining both NT‐proBNP and echo enhanced risk prediction accuracy (C‐index 0.813 vs 0.779). Additionally, reduced LVEF (<45%) and impaired GLS were independent predictors of cardiovascular events, underscoring the value of early echocardiographic evaluation for detecting subclinical myocardial impairment and improving prognosis in [88, 89]. Diastolic dysfunction is present in 60% of asymptomatic T2D patients without coronary disease [90]. T2D and impaired metabolism are common features in HFpEF, with >70% of HFpEF patients having T2D or pre‐diabetes [91]. A meta‐analysis confirms a pooled prevalence of over 50% in the broader T2D population [92]. GLS, measured via speckle‐tracking echocardiography, could be a highly sensitive tool for detecting subclinical systolic dysfunction in asymptomatic diabetic patients. In one key study, asymptomatic T2D patients had a significantly worse GLS (−17.8 ± 2.2%) compared to healthy controls (−20.2 ± 1.6%), despite having a normal ejection fraction. Furthermore, impaired GLS was a powerful predictor of future cardiovascular events, with a HR of 1.24 per 1% decrease, demonstrating its superior sensitivity over traditional EF for early risk stratification [93]. While directly comparative prevalence data between T1D and T2D are lacking, echocardiographic markers of diastolic dysfunction (e.g., *E*/*e*’ ratio) are strong predictors of adverse outcomes in T1D. This underscores the value of early echocardiographic assessment to detect subclinical dysfunction and guide timely risk management.

Routine early echocardiographic screening for all asymptomatic patients with T1D is not currently recommended by major societal guidelines (e.g., ADA), which advise a clinical, symptom‐driven approach. The role of advanced techniques like strain imaging for widespread screening in asymptomatic diabetic patients is an active area of investigation but is not yet standard of care [94].

Given the high prevalence of cardiovascular and microvascular complications in individuals with diabetes, comprehensive screening is essential. Accordingly, routine cardiovascular risk assessment in people with diabetes should include HbA1c measurement, blood pressure monitoring, lipid profile assessment, weight/BMI tracking, renal function tests, retinal screening, and foot examination. These diagnostic and preventive strategies support early detection of end‐organ damage and enable timely intervention, helping to reduce the burden of HF and its associated complications.

Optimal glycaemic control is a cornerstone of primary prevention, though its relationship with HF risk is complex. Evidence from a Scottish cohort demonstrates that poor glycaemic control, indicated by a higher HbA1c, is a strong predictor of HF hospital admissions specifically in T1D, accounting for a significant proportion of admissions [95]. However, intensive glycaemic control in older patients with long‐standing T2D and high cardiovascular risk has been paradoxically associated with an increased risk of HF hospitalization, highlighting the need for individualized targets [96].

Early cardiac assessment using biomarkers such as NT‐proBNP, a sensitive indicator of myocardial stress, is particularly valuable in people with T1D, as elevated levels above 125 pg/mL indicate myocardial stress and increased HF risk which enables early detection and treatment, potentially before symptoms occur [97, 98].

Lifestyle intervention is a cornerstone of HF prevention. The 2021 ESC guidelines recommend a healthy diet and sodium restriction (<5 g/day) for all patients at risk of HF. While plant‐based diets improve cardiometabolic risk factors, such as hypertension [1]. The ketogenic diet and SGLT2 inhibitor therapy, which increase ketone bodies, have sparked interest; however, the long‐term cardiac benefits in T1D require caution [99]. Blood pressure management, following standard guidelines, is critical for reducing afterload and preventing the development of HFpEF, a phenotype strongly linked to the hypertension often present in both T1D and T2D.

The PONTIAC trial demonstrated the efficacy of a biomarker‐guided, intensified preventive approach. In high‐risk diabetic patients (elevated NT‐proBNP) but no known heart disease, the initiation of RAAS inhibitors and beta‐blockers significantly reduced the primary endpoint of hospitalization or death from cardiac causes compared to standard care, providing a model for targeted primary prevention [100]. Despite this evidence, a major gap in care persists with the underutilization of cardioprotective drugs. Adherence to guideline‐directed medical therapy (GDMT) including ACE inhibitors/ARBs, and beta‐blockers is suboptimal in patients with diabetes. This is driven by barriers in T2D, such as polypharmacy, side effects, and lack of knowledge [101]. In T1D, additional factors including clinical inertia due to a historical focus on glycaemic control alone, a younger patient demographic, and a lack of trial data specific to T1D further contribute to this treatment gap, despite evidence that these agents reduce HF progression when used appropriately. Use of SGLT2 inhibitors and GLP‐1 receptor agonists offers additional cardioprotection in T2D and is being explored cautiously in T1D. Pharmacotherapy for secondary prevention and risk reduction has been revolutionized by new drug classes which are described in the next section.

## 5. HF Therapies in People With Diabetes

The contemporary management of HFrEF is anchored in the four pharmacological pillars, each targeting key pathophysiological pathways and supported by strong clinical evidence. These include of renin–angiotensin–aldosterone inhibitors (RAASi), beta blockers, mineralocorticoid receptor antagonists (MRAs), and sodium‐glucose cotransporter‐2 (SGLT2) inhibitors. Clinical guidelines recommend early initiation of these agents to improve survival and reduce hospitalizations in HFrEF patients. HF itself is an insulin‐resistant state, and evidence suggests that certain HF therapies may improve glycaemic control in patients with HF and diabetes, although this evidence is predominantly limited to T2D [102]. Figure 1 summarizes key comparative features and drivers of HF in T1D vs T2D.

Despite differing in pathophysiology, guideline‐directed therapy for HFrEF is largely consistent across T1D and T2D. Individuals with T1D are generally underrepresented in major HF therapy trials, often comprising only ~1% of enrolled diabetic participants. Consequently, it is assumed, but not proven, that T1D patients benefit from foundational HF therapies similarly to T2D patients, except for SGLT2 inhibitors. This is particularly notable for SGLT2 inhibitor HF trials, where people with T1D had been completely excluded, primarily due to concerns about diabetic ketoacidosis (DKA). This exclusion has contributed to a significant knowledge gap and therapeutic inequity for T1D populations, unlike T2D individuals who benefit from a growing body of comprehensive trial data [103, 104]. Recent cardiovascular outcome trials of novel antidiabetic agents, including SGLT2 inhibitors and GLP‐1 receptor agonists (GLP‐1 RA), have expanded HF treatment options for patients with T2D or without diabetes. However, these trials may further widen the evidence gap for T1D patients with HF.

RAAS inhibitors, beta‐blockers, and MRAs are foundational therapies for HFrEF. Most guidelines, however, do not recommend SGLT2 inhibitors for T1D. SGLT2i provide clinical benefits through mechanisms that are not yet fully understood but are believed to be multifactorial. Importantly, these benefits are independent of T2D status [105]. Across the spectrum of HF, SGLT2 inhibitors reduce HF hospitalization by ~28% and cardiovascular death by ~13% [106]. The exact mechanisms remain unclear. Likely contributors include systemic effects, such as osmotic diuresis and weight reduction, and direct cardiac actions, including favorable myocardial remodeling and metabolic changes [107, 108]. Individuals with T1D were excluded from major SGLT2i outcome trials due to the increased risk of DKA. SGLT2 inhibitors increase the risk of DKA in T1D, with real‐world incidence of ~ 20.2 events per 100 person‐years, independent of age, sex, or BMI. Risk factors include BMI >27 kg/m^2^, IR, rapid insulin dose reduction, and dehydration [109]. Appropriate patient selection and vigilant monitoring can help mitigate these risks. SGLT2 inhibitors have also been shown to improve glycaemic control and cardiovascular risk profiles in T1D [110]. In HFrEF patients with T2D, the combined use of all four foundational therapies was associated with a 73% relative risk reduction in all‐cause mortality, an NNT of 3.9, and a median survival extension of 7–11 years. In a 1‐year study of T1D, Sotagliflozin, combined with optimized insulin therapy, produced sustained HbA1c reductions of up to 0.41% at 24 weeks and 0.31% at 52 weeks, significant weight loss (−4.32 kg), and reduced insulin doses (bolus −15.63%, basal −11.87%). More patients achieved HbA1c <7% with Sotagliflozin (40.3% at 400 mg vs., 15.7% placebo). Treatment also resulted in fewer severe hypoglycemia episodes and improved patient‐reported outcomes, but with an increased risk of DKA (3.4%–4.2% vs., 0.4% placebo). In pooled data from the inTandem trials involving 2980 adults with T1D, Sotagliflozin (200/400 mg) significantly reduced predicted 5‐ and 10‐year CVD risk by 6.6% and 6.4%, respectively (*p*  < 0.0001), and reduced 5‐year risk of end‐stage kidney disease (ESKD) by 5.0% (*p* = 0.0003). Similar benefits were observed in individuals with BMI ≥ 27 kg/m^2^, suggesting Sotagliflozin may offer meaningful long‐term cardiorenal protection in T1D [111, 112].

SGLT2 inhibitors, when added to insulin therapy in T1D, have been shown to improve glycaemic control, reduce body weight, and lower insulin requirements. A meta‐analysis of 15 trials involving 7109 participants demonstrated improvements in HbA1c, fasting plasma glucose, daily insulin dose, body weight, and blood pressure, although benefits on glycaemic control may attenuate after 6 months. The therapy did not increase hypoglycemia or severe hypoglycemia but was associated with increased risk of genital tract infections and ketone‐related adverse events, including DKA [113]. A pooled analysis of the inTandem 1 and 2 studies confirmed a higher incidence of DKA with Sotagliflozin, an SGLT2/SGT1 dual inhibitor, compared with insulin alone, leading the European Medicines Agency to withdraw Zynquista (Sotagliflozin) in the EU due to safety concerns [114].

To address the current evidence, gap in HF management among individuals with T1D, we are conducting the SOPHIST trial, a UK‐wide, multicentre, double‐blind, placebo‐controlled study (NCT06435156). This trial aims to enroll 320 participants with T1D and HF, who will be randomized to receive either Sotagliflozin 200 mg daily or a matched placebo. The primary endpoint is the change in quality of life from baseline, as measured by the Kansas City Cardiomyopathy Questionnaire (KCCQ) clinical summary score at 16 weeks. An enhanced safety strategy is being used, incorporating continuous glucose monitoring (CGM), regular ketone testing, and increased participant education. The SOPHIST trial represents a crucial step toward generating high‐quality evidence for the safe and effective use of SGLT1/2 inhibition in this underrepresented population [115].

HFpEF poses a major clinical challenge, especially in patients with diabetes. In individuals with T2D, SGLT2 inhibitors like empagliflozin and dapagliflozin significantly reduce HF hospitalizations and improve symptoms, independent of glycaemic control. In the EMPEROR‑Preserved trial (*N*  =  5988; LVEF  >  40%), empagliflozin reduced the composite risk of cardiovascular death or HF hospitalization by 21% (HR 0.79; 95% CI 0.69–0.90; *p* < 0.001), with a 28% relative risk reduction in HF hospital admissions specifically, consistent across patients with and without diabetes [71, 116]. Similar to this DELIVER study involving 6263 participants, where dapagliflozin lowered the risk of HF progression or CV death by 18% in people with HFpEF and [117]. A real‑world longitudinal study in T2D people with HFpEF (*N * =  100) demonstrated that dapagliflozin significantly reduced myocardial fibrosis, with −3.5% change in ECV fraction versus −0.8% for placebo (*p* < 0.001); LV mass index declined by 8.2 vs. 2.1 g/m^2^, and 6MWT improved by 45 vs. 10 m (*p*  =  0.01) [118]. Again, individuals with T1D have largely been excluded from these HFpEF trials, and the elevated risk of DKA limits the use of SGLT2 inhibitors in T1D [119]. Therefore, non‑pharmacological strategies—including rigorous blood pressure control, weight management, and tailored exercise—remain central to managing HFpEF risk in T1D patients.

The safe implementation of SGLT2 inhibitors in T1D is critically dependent on advanced monitoring technologies. Consensus guidelines strongly recommend their use to mitigate DKA risk, which was the most frequent adverse event in a phase 3 trial of dapagliflozin, occurring in 4.0% of patients in the 10 mg group [120]. To proactively manage this risk, a review of adjunctive therapy underscores the utility of CGM and ketone sensing. Data indicate that early ketone detection can prevent progression to DKA, while CGM helps identify hyperglycemic trends that precede ketosis. In real‐world applications, adherence to a structured monitoring protocol involving daily ketone checks and CGM trend analysis has been shown to significantly reduce DKA episodes, supporting their role as essential components of a safe SGLT2i treatment framework [121, 122].

Traditional MRAs, such as spironolactone and eplerenone, have demonstrated efficacy in reducing cardiovascular events and hospitalizations in patients with HF and diabetes. However, their clinical use is often limited by an increased risk of hyperkalaemia and acute kidney injury (AKI). Recently, a novel non‐steroidal MRA, Finerenone, has emerged as a promising alternative, showing significant benefits in reducing diabetic kidney disease progression.

A pooled analysis conducted by Agarwal et al. [123] included ~13,000 patients and compared the effects of Finerenone with placebo. The cardiovascular composite endpoint included cardiovascular death, non‐fatal MI, non‐fatal stroke, or hospitalization for HF, while the renal outcome was defined as a sustained ≥57% decline in estimated glomerular filtration rate (eGFR) from baseline over at least 4 weeks. The incidence of cardiovascular events was significantly lower in the Finerenone group (12.7%) compared to the placebo group (14.4%). Furthermore, Finerenone demonstrated a significant protective effect on renal outcomes ([HR] 0.77; 95% [CI], 0.67–0.88; *p* = 0.0002), indicating a safer renal profile over a 3‐year period [123].

Following this, the ARTS‐HF trial, a randomized phase 2b study published in 2016 [124], evaluated Finerenone’ s cardiovascular effects in patients with HF and T2D or CKD. The trial showed that Finerenone was well tolerated and produced reductions in NT‐proBNP levels comparable to eplerenone [124]. The larger FIDELIO‐DKD trial [125] definitively demonstrated that Finerenone significantly reduced major adverse cardiovascular events (MACE), including fatal or nonfatal MI, stroke, cardiovascular death, and hospitalization for HF, with a HR of 0.77 for cardiovascular outcomes. Additionally, Finerenone slowed the decline in eGFR and reduced albuminuria, confirming its renal protective effects [125, 126]. These benefits were consistent regardless of patients’ baseline history of HF, highlighting Finerenone’ s dual cardiorenal efficacy over a median follow‐up of 2.6 years in people with T2D and CKD [127]. Similar trends were observed in the FIGARO‐DKD trial which assessed finerenone in 7437 individuals with T2D and diabetic kidney disease. Over a median follow‐up of 3.4 years, finerenone reduced the primary composite cardiovascular outcome, including cardiovascular death, nonfatal MI, nonfatal stroke, or HF hospitalization, by 13% versus placebo (HR 0.87; 95% CI 0.76–0.98; *p* = 0.03) [49]. This benefit was driven largely by a 29% reduction in HF hospitalizations (HR 0.71; 95% CI 0.56–0.90) [128].

The FINEARTS‐HF trial (2025) was a large international trial evaluating Finerenone in about 6000 patients with HF and preserved or mildly reduced ejection fraction (LVEF  ≥  40%). Over a median follow‐up of 32 months, Finerenone significantly reduced the composite primary outcome of total worsening HF events and cardiovascular death by 16% (rate ratio 0.84; 95% CI, 0.74–0.95; *p* = 0.007), including an 18% reduction in recurrent hospitalizations for HF (rate ratio 0.82; 95% CI, 0.71–0.94; *p* = 0.006) [129]. These data certainly support the use of nsMRA in HF patients, including those with diabetes (around 41% of the population had diabetes at baseline).

As is typical, most of these trials did not include T1D individuals, though there is no particular reason why nsMRAs should not be tolerated in T1D individuals. Only 15 individuals with T1D were included in FINEARTS‐HF. The FINE‐ONE trial (2023), a phase III, randomized, placebo‐controlled study is evaluating Finerenone in ~220 adults with T1D and CKD [130]. Over 7.5 months, the trial investigates the effect of Finerenone on the primary endpoint: relative change in urine albumin‐to‐creatinine ratio (UACR) from baseline at 6 months. UACR is used as a validated surrogate for kidney outcomes, based on prior T2D data. Secondary outcomes include safety measures such as hyperkalaemia and serious adverse events. If successful, FINE‐ONE could lead to the first approved therapy for CKD in T1D in nearly three decades and may pave the way for dedicated use in T1D individuals with HF.

Patients with T2D most frequently develop HFpEF recognized in DIABET‐IC Study (2025) as driven by a pathophysiology cantered on adipose‐driven systemic inflammation and lipotoxicity [131], whereas T1D, while also conferring risk for HFpEF via glucotoxicity, carries slightly higher risk for HFrEF often related to premature coronary artery disease [132, 133]. This phenotypic divergence means that the evidence base for HF therapies, including SGLT2 inhibitors, is predominantly derived from populations with high T2D prevalence. The efficacy of SGLT2 inhibitors themselves, however, is well‐established across the EF spectrum (HFrEF, HFmrEF, HFpEF) for patients with and without diabetes. The primary complexity in applying these therapies to T1D relates to safety management (e.g., DKA risk mitigation) and the absence of cardiovascular outcome trials specifically in T1D populations, rather than a lack of efficacy in a particular HF phenotype [134].

Current HF management guidelines do not differentiate between diabetes types, and most evidence is derived from studies in T2D populations, with an extrapolation of these recommendations to T1D. This underscores the lack of tailored guidance for T1Ds populations [25, 135]. Future research should integrate T1D patients into HF studies through registry‐based recruitment, stratified subgroup analyses, or dedicated trials, to ensure equitable representation and evidence‐based management [136]. Table 2 summarizes current pharmacologic treatment recommendations for HF subtypes (HFrEF, HFmrEF, HFpEF), highlighting differences based on diabetes status.

**Table 2:** Summary of guideline‐recommended key pharmacologic therapies for heart failure subtypes (HFrEF, HFmrEF, HFpEF) by diabetes types.

HF subtype	No diabetes	T2D	T1D
HFrEF (EF < 40%)	ACEi/ARB/ARNI (✔) BB (✔) MRA (✔) SGLT2i (✔)	ACEi/ARB/ARNI (✔) BB (✔) MRA (✔) SGLT2i (✔)	ACEi/ARB/ARNI (✔) BB (✔) MRA (✔) SGLT2i (X)
HFmrEF (EF 40%–49%)	SGLT2i (✔) ACEi/ARB/ARNI (△) MRA (△) BB (△) GLP‐1 RA (△ if overweight/obese)	SGLT2i (✔) ACEi/ARB/ARNI (△) MRA (△) BB (△) GLP‐1 RA (△ if overweight/obese)	SGLT2i (X) ACEi/ARB/ARNI (△) MRA (△) BB (△)
HFpEF (EF ≥50%)	SGLT2i (✔) ACEi/ARB/ARNI (△) MRA (△) GLP‐1 RA (△ if overweight/obese)	SGLT2i (✔) ACEi/ARB/ARNI (△) MRA (△) GLP‐1 RA (△ if overweight/obese)	SGLT2i (X) ACEi/ARB/ARNI (△) MRA (△) GLP‐1 RA (X)

*Note:* SGLT2 inhibitors and GLP‐1 receptor agonists are recommended in type 2 diabetes across all heart failure phenotypes due to cardiovascular benefits but are not recommended in type 1 diabetes because of safety concerns and limited evidence. Therapeutic options for T1D individuals with HFmrEF and HFpEF are particularly limited.

Abbreviations: ACEi, ACE inhibitor; ARB, angiotensin receptor blocker; ARNI, angiotensin receptor‐neprilysin inhibitor; BB, beta blocker; DKA, diabetic ketoacidosis; GLP‐1 RA, GLP‐1 receptor agonist; GLT2ii, Sodium‐glucose co‐transporter‐2 inhibitor; MRA, mineralocorticoid receptor antagonist.

✔ = Recommended.

△ = Consider.

X = Not recommended.

## 6. Diabetes Therapies in HF Patients

Metformin remains the preferred first‐line therapy for most patients with newly diagnosed T2DM and is considered safe in those with stable HF [137]. Evidence shows that metformin may reduce cardiovascular morbidity and mortality, and its cardioprotective effects may extend beyond glycaemic control, suggesting benefit even in nondiabetics [138]. The ongoing DANHEART trial is the first RCT powered to test whether these benefits translate into improved outcomes in patients with HFrEF and prediabetes or T2DM [139]. The data on metformin’s safety in HF are from T2D studies, most notably the UK Prospective Diabetes Study (UKPDS) 34 trial, which demonstrated cardiovascular risk reduction in overweight patients with newly diagnosed T2D [140]. There are no large‐scale, randomized trials specifically investigating metformin use for HF outcomes in patients with T1D or T2D. While the glycaemic efficacy of TZDs is comparable to metformin, adverse effects and increased HF risk make them less suitable as initial therapy, with placebo‐controlled trials showing that TZDs significantly and consistently increase the risk of both overall and severe HF [141]. In PROactive trial, pioglitazone was associated with an increased risk of HF hospitalizations and with macrovascular events [142]. Therefore, TZDs are contraindicated in HF population regardless of the specific agent, a recommendation upheld by major guidelines including the ADA [135]. Sulfonylureas have been consistently associated with increased cardiovascular risk, including a higher incidence of ventricular arrhythmias, sudden cardiac death, and HF, particularly at higher doses, compared to metformin, regardless of diabetes severity or coronary history. These risks seem to be a class effect but are amplified in patients with pre‐existing CVD, CKD, and advanced age, where the pro‐arrhythmic potential of hypoglycemia and the fluid‐retention properties of these drugs have the most severe consequences [143]. Insulin may worsen HF by promoting sodium retention, sympathetic activation, and weight gain [144]. Some studies suggest insulin use in HF is associated with increased risk of hospitalization and mortality [145], though of course it is mandatory in T1D. There is little evidence around the impact of newer technologies (e.g., hybrid‐closed loop, use of CGM, etc.) on HF. There are no randomized trials that have directly studied insulin delivery strategies for HF risk reduction in T1D. DCCT/EDIC study found that intensive therapy reduced the risk of CV event by 42% and non‐fatal MI, stroke, or CV death by 57% over 30 years of follow‐up [146]. Beck et al. [147] showing closed‐loop control that increased time‐in‐range (70–180 mg/dL) to over 70%, significantly reducing hyperglycemia. It is possible that advanced insulin technologies that optimize glucose management could reduce the risk of HF further, although more research is needed to confirm this [147].

Several large‐scale cardiovascular outcome trials have evaluated the efficacy of GLP‐1 RAs in people with T2D, overall demonstrating reductions in the incidence of atherosclerotic CVD such as non‐fatal MI, non‐fatal stroke, as well as cardiovascular death [148]. Beyond cardiovascular benefits, GLP‐1 RAs exert pleiotropic effects, including renal protection, blood pressure reduction via natriuretic peptide modulation, and significant weight loss. Additionally, GLP‐1 RAs are associated with reduced inflammation and improved endothelial function, further contributing to cardiovascular risk mitigation in people with diabetes [149]. The effects of GLP‐1 RAs on HF outcomes or in individuals with HF are less clear and the role of GLP‐1 RAs in patients with HF remains underexplored. Three small randomized trials have investigated GLP‐1 RAs in HFrEF populations: the LIVE trial, which examined the effects of liraglutide on left ventricular function over 24 weeks in patients with chronic HF (CHF); the FIGHT (Functional Impact of GLP‐1 for HF Treatment) trial, which assessed the impact of GLP‐1 RA therapy in 300 patients with HFrEF, with or without T2D; and a smaller trial evaluating albiglutide 30 mg versus placebo in patients with HFrEF and diabetes [150–152]. However, these studies did not demonstrate significant improvements in MACE reduction or LVEF and indeed suggested a potentially deleterious effect. Intriguingly, however, in recent data from a prespecified analysis of the SELECT trial, in which ~24% of the participants had pre‐existing HF, Semaglutide significantly reduced MACE by 20% (HR 0.80) and the composite HF endpoint (cardiovascular death or HF hospitalization) by 21% (HR 0.79). Notably, these benefits were consistent across subgroups with both reduced and preserved ejection fraction. Patients with HF at baseline experienced even greater benefit from Semaglutide, with HRs of 0.72 for MACE, 0.79 for the HF composite, 0.76 for cardiovascular death, and 0.81 for all‐cause mortality. These effects were independent of age, sex, BMI, NYHA class, and diuretic use. The findings suggest that the question of GLP1‐RA use in HFrEF patients with T2D remains open [153].

The evidence for GLP1‐RAs in HFpEF seems more compelling, although again large outcome trials are awaited. The STEP‐HFpEF DM trial evaluated once‐weekly Semaglutide 2.4 mg in patients with HFpEF and obesity with coexisting T2D. Semaglutide significantly improved HF‐related symptoms, physical limitations, and exercise capacity. The mean weight loss was 9.8% at 12 months, and reductions in CRP levels indicated improved systemic inflammation. These benefits were consistent across subgroups and were achieved with a safety profile like prior STEP trials. The trial highlights Semaglutide dual benefit in managing both metabolic and HFpEF‐related impairments in people with diabetes [154]. The SUMMIT trial investigated the dual GIP/GLP1‐RA Tirzepatide in patients with HFpEF and obesity. Results showed a 38% reduction in cardiovascular death or worsening HF events compared to placebo. Patients also experienced significant improvements in symptoms and physical function measured by the KCCQ‐CSS score. However, gastrointestinal side effects were more frequent, contributing to increased discontinuation rates [155]. Further insights into potential mechanisms of benefit were also obtained, with a sub study finding that Tirzepatide reduced LV mass and paracardial adipose tissue, suggesting some direct cardiac action [156].

GLP‐1 receptor agonists, although demonstrating cardiovascular benefits in T2D, have not been evaluated in large‐scale cardiovascular outcome trials in T1D. Smaller studies suggest potential reductions in body weight and insulin requirements, but their efficacy and safety in T1D remain less well established. Overall, these findings highlight that most cardioprotective recommendations in T1D are extrapolated from T2D trials, and dedicated outcome trials are limited.

Unfortunately, all this promising data excluded T1D individuals. The use of GLP‐1 receptor agonists (GLP‐1 RAs) in individuals with T1D mellitus (T1D) remains limited, with few clinical studies investigating their efficacy and safety. Critically, although there one large trial examining the use of GLP1‐RA in T1D individuals with a focus on CV outcomes, (Steno1: NCT06082063), there are none in T1D populations with HF, risking further widening the current evidence gap. The ongoing Steno‐1 trial (NCT06082063) is a pivotal cardiovascular outcomes trial for T1D. This randomized, placebo‐controlled study is evaluating oral semaglutide in ~3600 adults with T1D and high cardiovascular risk. Its primary endpoint is time to first MACE (cardiovascular death, non‐fatal MI, or non‐fatal stroke), with an estimated completion date of July 2029 [157].

## 7. Novel Therapies

To move beyond generalized cardiometabolic management, the next frontier in treating diabetic cardiomyopathy lies in developing novel, phenotype‐specific therapies. These strategies must directly target the distinct underlying drivers of myocardial injury in T1D and T2D, such as glucotoxicity‐driven fibrosis, adipose‐derived inflammation, and insulin/C‐peptide deficiency, to improve precision and efficacy.

C‐peptide has been redefined from an inactive byproduct to a potent, endogenous hormone with significant therapeutic potential for diabetic microvascular complications. Clinical trial evidence supports its role, showing that C‐peptide replacement can significantly reduce urinary albumin excretion—a key marker of nephropathy—by 30%–40% and improve peripheral nerve function in patients with T1D. Its mechanisms, including activation of Na^+^/K^+^‐ATPase, stimulation of NO production (enhancing microvascular blood flow by 20%–25%), and anti‐inflammatory effects, directly target the pathophysiology of neuropathy and nephropathy. The development of long‐acting analogs like once‐weekly Ersatta positions C‐peptide replacement therapy as a promising disease‐modifying strategy, offering a new avenue to improve outcomes in T1D and, potentially, insulin‐deficient T2D [157, 158].

The CANTOS trial confirmed the link between inflammation (IL‐1β/CRP) and worse cardiometabolic outcomes but revealed a critical nuance. Targeting inflammation with canakinumab significantly reduced HF hospitalizations in a dose‐dependent manner, with the 300 mg dose showing a HR of 0.76 (95% CI: 0.57–1.01) for HF hospitalization [159]. However, the same anti‐inflammatory therapy did not reduce the incidence of new‐onset T2D (HR for all doses vs., placebo: 1.02; 95% CI: 0.87–1.19), despite transiently lowering [160]. This suggests that while inflammation is a key driver of HF progression in established CVD, it may not be the primary instigator of metabolic dysfunction leading to diabetes itself. Therefore, anti‐inflammatory strategies like canakinumab hold promise for secondary HF prevention in high‐risk patients but not for primary diabetes prevention.

Pirfenidone is an oral antifibrotic drug with potential as a novel therapy for diabetic cardiomyopathy, specifically targeting the pathological myocardial fibrosis central to its progression. Its mechanism involves inhibiting key profibrotic and inflammatory mediators; in cardiac fibroblasts, pirfenidone can reduce TGF‐β1‐stimulated collagen synthesis by up to 50% [161]. While preclinical animal models of diabetic cardiomyopathy show it can reduce left ventricular fibrosis by ~30% and improve diastolic function, human evidence is emerging but limited [162]. The recent positive Phase II PIROUETTE trial in HFpEF patients, a population with high diabetic comorbidity, demonstrated its ability to significantly reduce cardiac fibrosis as measured by a −1.21% absolute reduction in ECV on CMR versus placebo. However, as a pleiotropic agent, definitive evidence from large‐scale, outcome‐driven clinical trials in diabetic populations is still needed to establish its safety and efficacy as a standard treatment.

## 8. Conclusions

Advances in blood glucose monitoring and insulin delivery, which have increased life expectancy, are expected to make HF an increasing clinical burden in T1D. Existing data already highlight disparities in HF outcomes between T1D and T2D individuals, with younger T1D individuals being particularly vulnerable. Absolute HF incidence is higher in T2D. However, the relative risk of HF versus non‐diabetics is equally or more pronounced in T1D, especially in older age groups, highlighting a serious and potentially underappreciated burden. As more therapies become available for HF, this gap may widen unless clinical research actively includes T1D individuals. Therefore, generating high‐quality, targeted evidence is urgently needed to ensure timely and equitable treatment strategies for individuals with T1D and HF. Two key issues need to be addressed: (1) The lack of inclusion of T1D individuals in HF trials and (2) Identification of the specific differential pathophysiology between HF in T1D and other groups, and development of novel strategies to target these. As the therapeutic landscape for HF continues to expand, the disparity in clinical outcomes is likely to widen unless individuals with T1D are actively included in future research. Future health economic analyses must prioritize LMIC perspectives to inform sustainable, context‐specific strategies. Ultimately, implementing existing cost‐effective policies and interventions is urgently needed to meet the targets for Sustainable Development Goal 3 (SDG3) and achieve a 30% reduction in premature mortality from noncommunicable diseases. The paucity of T1D participants in HF trials also underscores the urgent need for robust, dedicated studies enrolling exclusively T1D patients to establish evidence‐based, tailored management strategies. The evaluation of novel pathophysiology‐specific therapies, such as dual GIP/GLP‐1 receptor agonists and agents targeting coronary microvascular dysfunction, is crucial to improve outcomes in this high‐risk population. This evolving paradigm underscores the future need for precision medicine strategies in diabetic cardiomyopathy, integrating advanced biomarkers, myocardial strain imaging, and genetic profiling to differentiate HF phenotypes and tailor pathophysiology‐specific therapies. Clinicians should remain vigilant for cases of subclinical HF in T1D, incorporating regular assessment of cardiovascular risk factors with NT‐ProBNP detection and early echocardiographic evaluation, even in the absence of overt symptoms.

## Funding

Ify R Mordi is supported by a British Heart Foundation Intermediate Clinical Research Fellowship (FS/ICRF/24/26101). Yi Jia Liew is supported by a Breakthrough‐T1D Strategic Research Agreement (3‐SRA‐2023‐1376‐M‐B).

## Conflicts of Interest

The authors declare no conflicts of interest.

## Data Availability

Data sharing not applicable to this article as no datasets were generated or analysed during the current study.
